# The changing face of farmers’ home gardens: a diachronic analysis from Sillian (Eastern Tyrol, Austria)

**DOI:** 10.1186/s13002-018-0262-3

**Published:** 2018-10-29

**Authors:** Brigitte Vogl-Lukasser, Christian R. Vogl

**Affiliations:** Division of Organic Farming, Department for Sustainable Agricultural Systems, University of Natural Resources and Life Sciences, Vienna, Gregor-Mendel-Straße 33, 1180 Vienna, Austria

**Keywords:** Gardening, Garden, Subsistence, Ethnobotany, Agrobiodiversity, Land use change, Transition, Sustainability, Alps, Mountain farming, Agroecology, Homegarden

## Abstract

**Background:**

Home gardens are an integral part of many traditional land use systems around the world. They are subject to various conversion processes and undergo a variety of changes. We were interested if change is an ongoing process in farmers’ home gardens of Eastern Tyrol (Austria).

**Methods:**

In Sillian, 16 farmers’ home gardens (FHGs) were studied. They had been studied in 1998 and were revisited in 2013 including again a botanical inventory of cultivated and non-cultivated plants, and structured interviews on appearance, management and plant use. In 2017, all the 16 gardens were visited again to verify whether any visible change on spatial configuration had occurred.

**Results:**

The home garden size had decreased between 1998 and 2013. A wider range of sizes was observed. The occurrence of plant taxa per garden was the same but an increase in the standard deviation of occurrence is seen. Plant diversity (occ./m^2^) increased between 1998 and 2013. Seventy-nine plant taxa were no longer cultivated in 2013, but 95 new plant taxa were being cultivated. The correlation between garden size and occurrence was not significant, i.e. small gardens might host many different plant taxa or large gardens might have fewer plant taxa. The occurrence for certain use categories was not significantly different between the years, except for the increase in the occurrence of plant taxa used as food and the food subcategory spice. The mean abundance of individuals for all plant taxa showed a significant decrease between the years. In 2013, an increase in standard deviation of abundance is seen. The variation in the different use categories expressed in abundance between the years was not significantly different, except for the decrease in the abundance of plant taxa used as food. Between 1998 and 2017, six home gardens showed a change of their spatial configuration (replacement by raised beds; merging with other structures; conversion to lawn). One FHG shows signs of abandonment.

**Conclusions:**

In Sillian, gardens are by no way static agroecological units, but are dynamic and individual in their appearance, composition and function. Farmers’ home gardens in Sillian show a trend towards becoming more individual, i.e. conversion from being a product of a homogenous local cultural script of the community into an area where gardeners define more individually the role that farmers’ homegardens are expected to play for them or their family.

## Background

Home gardens are an integral part of many traditional land use systems around the world [1–5]. In Europe, traditional land use systems focusing on subsistence and the local exchange of produce have seen dramatic changes in recent decades as a result of various socio-economic transformations that are also perceived to be a threat to the continuation of diversified agroecosystems [6–9].

The second half of the twentieth century is often identified as the period in which agriculture shifted from a “traditional”, subsistence-oriented agrarian mode of production to a “modern”, commercially oriented one [10–12].

In Eastern Tyrol, that period saw the abandonment of arable subsistence farming (e.g. the production of rye and wheat) in the mountainous parts of the region and many labour-intensive manually operated subsistence practices (e.g. the gathering of wild plants for food/medicinal purposes [13, 14]), as was the case in other parts of Europe as well [11, 15]. Decisions were taken in Eastern Tyrol to specialise in grassland with higher livestock productivity and a strong focus on national and international markets [11, 16].

There have also been changes in farmers’ home gardens (FHGs) in Eastern Tyrol. Until the 1960s, FHGs were small, fenced, horticulturally managed plots with limited plant diversity focusing on medicinal use and spices. In 1998, these FHGs had increased in their diversity, size and importance for subsistence, with some plant taxa even entering the garden from fields in distinct environments [17, 18], as also observed for example by Coomes and Ban [19], showing that FHGs are a dynamic system [17, 20]. Indeed, home gardens are not only recognised worldwide as multi-purpose, ecological and socially sustainable systems [1–3, 21], but also as exhibiting changes over time in line with the needs and views of those managing these agroecosystems [2, 17, 22].

As dynamic systems, home gardens are subject to various conversion processes worldwide and undergo a variety of changes—garden modernisation does not occur uniformly [23]—with regard to plant diversity, functional diversity, structural diversity, knowledge and resource inputs for example [17, 24–27]. Changing cultural values, socio-economic, demographic, political and climatic conditions, plus technical and infrastructural development—among other variables—are mentioned as underlying driving forces of this [2, 17, 25, 28–30].

The collection of data at different time periods could open up new perspectives for understanding the dynamics of the home garden system [3, 25, 31]. FHGs in Eastern Tyrol were studied in 1998 with regard to their dynamics from the 1960s to their status in 1998. The present study analysed the dynamics of the management, structure, plant use (occurrence and abundance) and function of FHGs in Sillian (Eastern Tyrol) between 1998 and 2013. Furthermore, data from 2017 were also studied to identify whether FHGs continued to be production systems.

## Methods

### Study area

The district of Lienz (Eastern Tyrol) is located in the Austrian part of the Eastern Alps. The large altitudinal gradient from 600 m to almost 4000 m above sea level gives rise to a narrow sequence of different natural and agricultural zones. Annual precipitation in the region is 826–1354 mm, and the mean annual temperature is 2.8–6.9 °C (values depend on exposure and altitude). This broad range of natural conditions within a small area has led to a highly diverse pattern of human-environment relationships [32]. Adaptive management of natural resources by Alpine small farmers has created a typically diverse and multifunctional landscape. The historical form of agriculture in this region can be described as “mountain cereal grazing” [33] in which the farming of arable land (up to 1700 m a.s.l.) for cereal cultivation, field vegetables, fibre crops, etc. and the farming of a wide range of domesticated animals, with a low number of individuals per species, were the main components of the subsistence system until the 1960s [13, 17, 34]. Large parts of today’s meadowlands used to be tilled up to an altitude of 1700 m. Farming systems in Eastern Tyrol have undergone change in the past few decades. The cultivation of cereals, fibre crops and field vegetables (e.g. *Pisum sativum* L., *Vicia faba* L., *Brassica rapa* L. ssp. *rapa*) has declined in the last three decades due to unfavourable economic conditions and the need for high labour inputs. The agricultural focus of mountain farms today is on grasslands, with the cultural landscape dominated by meadowland in lower zones, where hay is produced for winter fodder, and by pastureland in the higher alpine zones, where cattle remain throughout the summer. The economy of the majority of mountain farms in Eastern Tyrol is based on cattle breeding, milk production and timber harvesting for cash income. Some farmers offer beds to tourists and/or process milk, meat and other products from the farm. For their own consumption, some farmers diversify their basic activities by also keeping sheep, goats, pigs, chicken or bees, and/or growing fruit, herbs and vegetables (e.g. potatoes). Farming is combined with different kinds of off-farm labour, with federal subsidies playing an important role in farm income [13].

The village of Sillian, one of the 33 villages in Eastern Tyrol, is situated at 1100 m.a.s.l. (village centre) in the Pustertal valley in the western part of the district of Lienz. The village includes various hamlets, such as Sillian Berg and Arnbach, and has a population of 2044 in total. In this village, 124 farms manage a total of 2964 ha, an area that is constantly declining in parallel with a decreasing number of farmers working the land [35–39].

### Data collection and analysis

Farmers’ home gardens, according to the local perception of the term *Bauerngarten* used in this area, are small, manually operated horticultural cultivation spaces adjacent to the farmers’ households, in which annual, biennial and perennial cultivated plants are grown (also following the definition of the previous study in 1998 [13]). In 1998, FHGs were perceived by the gardeners and the authors of that study as discrete units with a determinate boundary (called “FHG with traditional spatial configuration”), easily identified as a unit used entirely for horticulturally cultivated plant taxa and clearly distinct from, for example, arable plots, orchards, pure ornamental plots in front of the house or recreational areas close to the homestead [18]. Arable land (e.g. planted with potatoes) where ploughing is characteristic, orchards where fruit trees (e.g. apple) are a dominant feature or gardens where only ornamental plants are grown were not included in the data on FHGs presented here.

In this paper, cultivated plant taxa refer to domesticated plants and wild plants under incipient management (tolerated, encouraged or protected) [40] and the family member primarily responsible for managing the garden is called the “gardener”.

In 1998, 196 FHGs in 12 villages in Eastern Tyrol, including Sillian, were studied [17, 18, 20, 41]. In 2013, out of this sample of 12 villages, one village (Sillian) was selected for a comparative study and 16 FHGs here, corresponding to 16 farm households (*n* = 16), were investigated. Every garden recorded in 1998 was revisited in 2013. In 1998, the average age of the gardeners was 49, in 2013 53 years of age, with no statisticalyl significant difference. We do refer to specific gardens by adding to the abbreviation FHG our internal respondents’/Garden ID such as e.g. “FHG_1011”. In two cases where the garden had been relocated, the new location was studied. In Sillian in 2013 a total of 2060 inhabitants were living. The village counts with 67 farms. To our observation all these farms do have FHGs. Therefore, the sample of 16 FHGs represents roughly a quarter of all FHGs of Sillian.

The reasons this village was sampled were the typicality and representativeness of the village, its hamlets and FHGs in Eastern Tyrol. Sillian is a village with FHGs in the urbanised valley plain, but also along a gradient of altitude up to 1573 m.a.s.l., and along a gradient of distance with FHGs also situated in remote valleys—comparable to Eastern Tyrolean villages such as Matrei or Virgen for example.

In 1998 and 2013, a botanical inventory of cultivated and non-cultivated, i.e. spontaneously reproducing plants [41], was undertaken. In 1998 this was done during three visits in the growing season in early May, July and October, while in 2013 the inventory was conducted in midsummer (July). Therefore plants only grown in the spring and autumn of 1998 were excluded from the comparative evaluation. For the botanical inventories in 1998 and 2013, wherever possible a sterile or fertile plant voucher specimen was collected and added to the authors’ collection and deposited in the herbarium at the University of Natural Resources and Life Sciences in Vienna. Plants were identified mainly in the field or in the laboratory based on the collected vouchers, as also described by [42].

Structured interviews were conducted with each responsible gardener. Among other topics, these interviews collected information on the appearance and management of FHGs and on plant use. Furthermore in 2013 the person responsible for the garden (the gardener) was interviewed about the changes observed in the recent period since 1998.

In 2017 all the FHGs were visited once to be photographed and checked to verify whether it was still possible to identify the FHGs at the homestead and if any visible changes on spatial configuration—compared to 2013—had occurred. No interviews, inventory or measurements were conducted in 2017.

The nomenclature used in this paper is in accordance with [43]. Cultivated plants are aligned with the official nomenclature of the International Code for the Nomenclature of Cultivated Plants (ICNCP), as acknowledged also by the International Code of Nomenclature for algae, fungi and plants (ICBN). Inventoried plants were identified at genus, species (sp.), subspecies (ssp.) or varietas (var.) taxonomical levels. Popular garden plants were summarised under the term Cultivars (e.g. all six different Rosa hybrid categories from 1998 were summarised as just *Rosa* L. Cultivars) or Groups (e.g. *Allium cepa* L. *Aggregatum* Grp.). Some names used in 1998 are no longer valid and these plants were renamed. In the present paper, all the ranks mentioned are referred to below as “plant taxa”.

Whenever possible and permitted, photographs were taken and deposited at the University of Natural Resources and Life Sciences in Vienna. Collected data were stored and categorised in an MS ACCESS (Microsoft Inc. 2013) database and subsequently analysed with SPSS (IBM SPSS Statistics 24).

The impact of the independent nominal variable year (1998 or 2013) on the dependent metric variables, such as number of species or number of individuals, was tested with a paired t-test. Correlations between garden size and other metric variables were tested using the Pearson correlation. A level (alpha) of < 0.05 was used when referring to significance and ≥ 0.05 to < 0.1 when referring to a tendency. Due to the small sample size, no tests were performed for the frequency of nominal variables (e.g. on management practices).

In the present analysis the parameters studied were defined as follows:

- Occurrence: the absolute frequency of plant taxa per FHG;
- Abundance: the absolute frequency of individual plants per plant taxon per FHG;
- Relative occurrence: the occurrence of plant taxa per FHG compared to total occurrence of plant taxa in the sample “*n*” (sum of plant taxa per garden divided by sum of all plant taxa in all gardens × 100, expressed in %);
- Relative abundance: the abundance per FHG compared to total abundance in the sample “*n*” (sum of individual plants per garden divided by all individual plants in all gardens × 100, expressed in %);
- FHG plant diversity: occurrence per m^2^;
- FHG abundance: abundance per m^2^;
- Functional characteristics: occurrence/abundance of different plant-use categories (ornamental, food, fodder, human medicinal, veterinary medicinal, fence, customs, fertiliser).

## Results

### Management

In 2013, all 16 gardeners were still managing their FHG in Sillian, compared to 1998. In 2013, the FHGs were still located right next to the farmhouses (mean distance: 1998, 10 m; 2013, 13 m). Fourteen FHGs were located on the same spot as that identified in the 1998 survey. Two FHGs had been relocated to another, steeper area because the flat areas of the former FHGs were needed for a path and a parking area respectively.

All 16 gardeners in 1998 were female, and of these, 11 were still managing the garden in 2013. Of the five successors, one was male. In 2013, 15 FHGs had a single gardener who was primarily responsible for the FHG (16 in 1998). One FHG was managed by several family members (not all of them lived in the household) and a neighbour due to the advanced age of the previous responsible gardener (FHG_1011). The most important motivation of gardeners in both years was an appreciation for home-grown produce. Of the 16 gardeners surveyed in 2013, 14 said that they would continue to manage the FHGs in future. Of these, three stated that they were enthusiastic gardeners and even wanted to expand their gardening. Ten gardeners wanted to continue gardening at the same level. One gardener was undecided (FHG_1012), and two gardeners had thought about giving up gardening to avoid the related workload (among them the male gardener; FHG_1008 and FHG_1003).

A change in equipment and supplies was rarely observed. Labour had not been replaced by machinery. In 2013, manual digging of the soil and manual weeding were still common. Synthetic pesticides were used by one gardener in 1998 and by three gardeners in 2013. All the gardeners interviewed used manure from their farm’s own cattle. Synthetic fertilisers were not used. The comparison between the years showed a higher frequency in 2013 of the use of homemade herbal teas for spraying plants, composting and mulching, of the use of green manure, the presence of recreational areas and raised beds within the FHG area, and a lower frequency of intercropping within beds for example. These differences were not statistically tested however due to the small number of cases.

### Size and spatial configuration

In 1998, 14 FHGs were separated from the surrounding area by fences; in 2013, this number was 12.

The FHG size (arithmetic mean; 1998, 65 m^2^; 2013, 48 m^2^; *p*_*t* test paired_ = 0.056) had decreased (tendency) between 1998 and 2013 (significant difference, if outlier FHG_1011 was excluded: 65 m^2^ in 1998; 42 m^2^ in 2013; *p*_*t* test paired_ = 0.006) (Table 1).

**Fig. 1:**
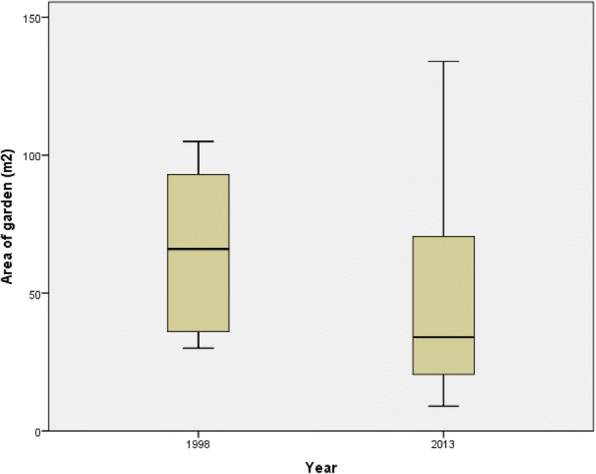
Garden size in square meters for *n* = 16 in 1998 and the same *n* = 16 in 2013, shown as a box plot with median

A wider range of FHG sizes (1998: min, 30 m^2^/max: 105 m^2^) in 2013 (min, 9 m^2^/max, 134 m^2^) was observed, increasing from 75 to 125 m^2^. In 2013, 13 FHGs showed a similar or smaller size, and three (FHG_1015, FHG_1011, FHG_1005) had increased their size, one of them (FHG_1011) from 72 to 134 m^2^ (Fig. 1, Table 1).

FHG_1011 was exceptional because it was the only FHG that had almost doubled in size by 2013. This gardener, and the family members who worked with her, had expanded the cultivated area by placing various small, horticulturally cultivated plots outside the former boundaries of the garden in an adjacent orchard. By reducing high-maintenance vegetable growing undertaken in 1998 to various small cultivated plots and expanding the growing of less demanding shrubs (e.g. redcurrant *Ribes rubrum* L.), a clearly delimited “low maintenance home garden space” was established, even though it was twice the size. Family members said that garden management was difficult for the responsible gardener due to her advanced age and was only feasible with the assistance of family members, although there was no explicit mention of her giving up gardening altogether.

In another case (FHG_1015), the garden size was slightly increased in 2013 by expanding the cultivated area across the former boundary and merging it with other structures (grassland) (Fig. 7).

In one case (FHG_1005), in 2013 the size had been increased by constructing an additional new garden with a traditional spatial FHG configuration. Respondents mentioned the estimation for self-supply with home-grown products and being passionate about gardening as reasons for the increase in size. These two gardeners along with FHG_1004 (similar size in both years) referred to themselves as “keen gardeners”.

For 2013, the reduction in the size under cultivation was achieved by converting horticulturally cultivated area into lawn for use as a play area for example. In three cases this led to a reduction of the clearly delimited cultivated area by ≥ 50% (FHG_1003, FHG_1007 and FHG_1010) (Fig. 7). In two cases, former FHGs were relocated, reducing the size of the new garden (FHG_1001, FHG_1012), but maintaining the traditional FHG spatial configuration. According to the respondents, the reasons for reducing the area of cultivation were the high labour demand (time that needed to be invested) for gardening, the perception of gardening as hard work or the reduced need for garden produce.

### Flora and function

#### Occurrence (number of plant taxa) and plant use

The total number of cultivated plant taxa found in the 16 inventoried home gardens in Sillian was 223 in 1998 and 239 in 2013 (Table 2).

**Fig. 2:**
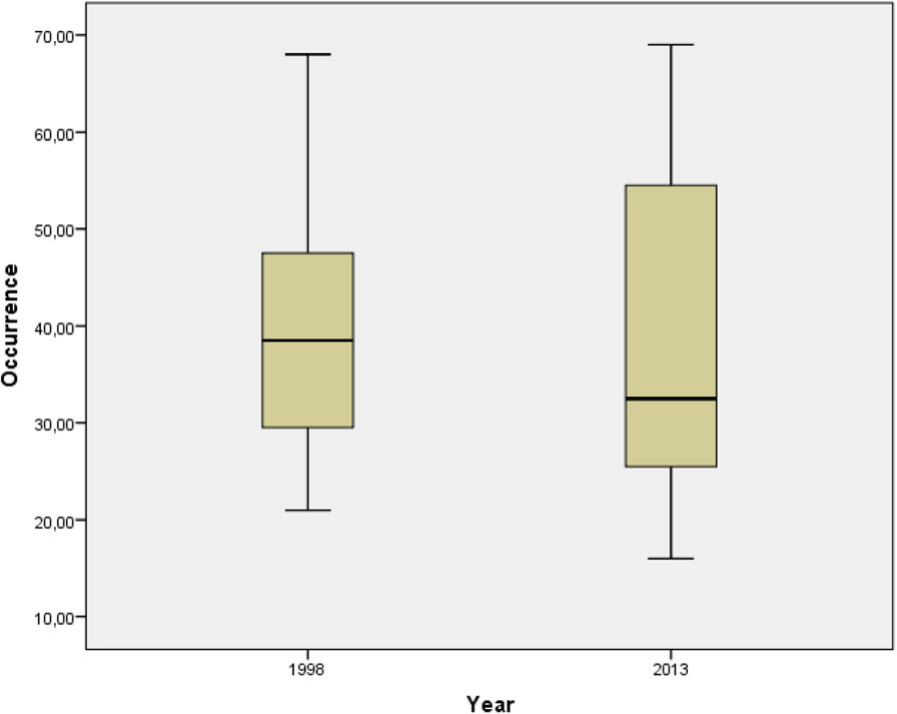
Presence of plant taxa (occurrence) for *n* = 16 in 1998 and the same *n* = 16 in 2013, shown as a box plot

There was no significant difference in the occurrence of plant taxa per FHG between the years (arithmetic mean; 1998, 39; 2013, 40; *p*_*t* test paired_ = 0.799) (Table 1). In 2013, an increase in variability (e.g. expressed in the standard deviation) could be seen (Table 1), i.e. gardens with a higher maximum and lower minimum number of plant taxa per garden compared to 1998 (Fig. 2).

One-hundred and six (1998) and 113 (2013) plant taxa were found in one garden alone. Seventy-nine plant taxa were no longer cultivated in the gardens in 2013 (compared to 1998). For example, the cultivation of *Cosmos bipinnatus* Cav., *Centaurea cyanus* L. and *Vicia faba* L*.* had been abandoned. In 2013, 95 new plant taxa were being cultivated, such as rocket salad (*Eruca sativa* Mill*.*) and basil (*Ocimum basilicum* L.).

In 1998, 20 plant taxa, and in 2013, 16 plant taxa contributed to the similarity (presence in ≥ 50% of FHGs) (Table 3), e.g. *Allium schoenoprasum* L. var*. schoenoprasum* was present in 100% of FHGs in both years. Eleven plant taxa were present in ≥ 50% of FHGs in both years.

**Table 1:** Overview of quantitative data obtained from *n* = 16 FHGs in 1998 and 2013

Year	1998	2013	*p*
Garden size			
Mean	65.56	48.13	*p* = 0.056
Median	66	34	
Std. deviation	28.467	37.352	
Minimum	30	9	
Maximum	105	134	
Range	75	125	
Occurrence			
Mean	39.19	40.00	*p* = 0.799
Median	38.50	32.50	
Std. deviation	12.55	16.90	
Minimum	21.00	16.00	
Maximum	68.00	69.00	
Range	47.00	53.00	
Abundance			
Mean	691.75	487.13	*p* = 0.027
Median	681.00	335.00	
Std. deviation	310.23	329.14	
Minimum	220.00	87.00	
Maximum	1138.00	1196.00	
Range	918.00	1109.00	
Plant use characteristics (occ.)	Min/Max/Mean	Min/Max/Mean	
Ornamental occurrence	1/31/17.13	2/40/18.56	*p* = 0.880
Ornamental abundance	14/301/118.50	6/414/115.50	*p* = 0.492
Food occurrence	6/33/16.63	10/53/23.25	*p* = 0.002
Food abundance	157/1007/506.94	86/1029/398.63	*p* = 0.047
Spice occurrence	1/16/6.19	1/22/7.88	*p* = 0.137
Spice abundance	34/821/170.69	7/420/125.44	*p* = 0.138
Customs occurrence	0/5/0.81	0/21/2.5	*p* = 0.147
Customs abundance	0/25/2.19	0/62/11.81	*p* = 0.042
Fertiliser occurrence	0	0/12/0.94	*p* = 0.189
Fertiliser Abundance	0	0/314/21.63	*p* = 0.205
VetMed occurrence	0/2/0.63	0/10/0.81	*p* = 0.029
VetMet abundance	0/102/11	0/130/10.19	*p* = 0.186
Fodder occurrence	0/3/0.31	0/1/0.31	*p* = 0.792
Fodder abundance	0/102/9	0/20/3.50	*p* = 0.322
Human medicinal occurrence	0/9/2.63	0/39/5.88	*p* = 0.121
Human medicinal abundance	0/25/2.19	0/62/11.81	*p* = 0.782
Occurrence per area			
Mean	0.7135	1.203	*p* = 0.021
Median	0.6236	1.1937	
Variance			
Std. deviation	0.39	0.72	
Minimum	0.31	0.25	
Maximum	1.63	2.78	
Range	1.33	2.53	
Abundance per area			
Mean	11.31	12	*p* = 0.700
Median	10.75	10.81	
Variance	21.08	30.03	
Std. deviation	4.59	5.45	
Minimum	4.67	3.37	
Maximum	19.39	22.31	
Range	14.72	18.95	
Relative occurrence			
Mean	17.71	16.73	*p* = 0.536
Median	17.26	13.6	
Std. deviation	5.63	7.07	
Minimum	9.42	6.69	
Maximum	30.49	28.82	
Range	21.08	22.18	
Relative abundance			
Mean	6.25	6.25	*p* = 1.000
Median	6.16	4.23	
Std. deviation	2.8	4.22	
Minimum	1.99	1.12	
Maximum	10.28	15.35	
Range	8.29	14.23	

The mean of the relative occurrence of plant taxa per garden was 17% for both years (no significant difference between the years; p_t-test paired_ = 0.536), with gardens containing between 7% and 30% (Table 1) of all plant taxa identified (FHG_1015; FHG_1002; FHG_1004 were the top three in both years).

**Table 2:** List of plant taxa identified in n= 16 FHGs in 1998 and 2013

Scientific name	Englisch name	Abundance98	Occurrence98	Abundance13	Occurrence13
*Acer platanoides* L.	Norway Maple	0	0	1	1
*Achillea filipendulina* Lam.	Fernleaf Yerrow	0	0	1	1
*Achillea millefolium* L.	Common Yerrow	0	0	3	1
*Achillea ptarmica* L.	Sneezewort	10	4	14	3
*Aconitum napellus* L.	Garden Monkshoot	0	0	1	1
*Aegopodium podagraria* L.	Ground Elder	0	0	100	1
*Agastache foeniculum* (Pursh) Kuntze	Anise Hyssop	1	1	0	0
*Agrostemma githago* L.	Corn Cockle	5	1	0	0
*Alcea rosea* L.	Hollyhock	0	0	20	2
*Alchemilla* L. sp.	Lady's Mantle	0	0	1	1
*Allium cepa* L. Aggregatum Grp.	Shallot	24	1	0	0
*Allium cepa* L. Cepa Grp.	Onion	710	11	428	8
*Allium fistulosum* L.	Welsh Onion	6	1	13	2
*Allium porrum* L. var. p*orrum*	Leek	151	9	204	11
*Allium ramosum* L.	Fragrant Garlic	1	1	0	0
*Allium sativum* L. var. *sativum*	Common Garlic	110	3	0	0
*Allium schoenoprasum* L. var. *schoenoprasum*	Chives	521	16	342	16
*Allium tuberosum* Rottler ex Spreng.	Oriental Garlic	0	0	11	2
*Allium ursinum* L.	Wood Garlic	0	0	6	2
*Althaea officinalis* L.	White Mallow	6	6	5	4
*Amaranthus caudatus* L.	Love Lies Bleeding	3	1	0	0
*Anacyclus* L. sp.	Anacyclus	1	1	0	0
*Anemone coronaria* L.	Windflower	0	0	3	1
*Anethum graveolens* L. var. *hortorum* Alef.	Dill	3	2	0	0
*Antirrhinum majus* L.	Snapdragon	41	4	17	3
*Apium graveolens* L. var. *rapaceum* (Mill.) Gaudin	Turnip -rooted Celery	100	8	41	6
*Apium graveolens* L. var. *secalinum* Alef.	Chinese Celery	16	2	0	0
*Aquilegia* L. Cultivars	Columbine	10	5	1	1
*Aquilegia vulgaris* L.	Columbine	0	0	19	4
*Armoracia rusticana* P.Gaertn., B.Mey. et Scherb.	Horseradish	7	5	47	3
*Arnica montana* L.	Mountain Arnica	0	0	6	1
*Artemisia abrotanum* L.	Southernwood	2	2	1	1
*Artemisia absinthium* L.	Common Wormwood	3	3	10	4
*Artemisia dracunculus* L.	Tarragon	3	2	1	1
*Artemisia vulgaris* L.	Mugwort	1	1	1	1
*Aruncus dioicus* (Walter) Fernald var. *dioicus*	Goat's Beard	1	1	0	0
*Asparagus officinalis* L.	Sparrow Grass	8	5	3	2
*Aster amellus* L.	Italian Aster	2	1	0	0
*Aster cordifolius* L.	Blue Wood Aster	1	1	2	1
*Aster dumosus* L.	Bushy Aster	5	4	0	0
*Aster novae-angliae* L.	New England Aster	8	2	8	2
*Aster novae-belgii* L.	Michaelmas Daisy	3	2	20	4
*Aster tongolensis* Franch.	Aster	1	1	0	0
*Astilbe* Buch.-Ham. Ex D. Don sp.	False Buck's Beard	2	1	0	0
*Aubrieta* Adans. Cultivars	Aubrietia	2	2	1	1
*Bellis perennis* L.	Daisy	70	1	16	2
*Beta vulgaris* L. ssp. *vulgaris* var. r*apacea* K.Koch	Beet	60	1	0	0
*Beta vulgaris* L. ssp. *vulgaris* var. v*ulgaris*	Beetroot	476	12	166	7
*Beta vulgaris* ssp. *cicla* (L.) W.D.J. Koch var. *cicla*	Foliage Beet	27	4	23	3
*Borago officinalis* L.	Borage	7	1	36	3
*Brassica napus* L. ssp. *rapifera* (Metzg.) Sinskaya	Swedish Turnip	40	1	0	0
*Brassica oleracea* L. var. b*otrytis* L.	Cauliflower	221	8	9	3
*Brassica oleracea* L. var. *capitata* (L.) Alef.	Cabbage	378	11	137	7
*Brassica oleracea* L. var. *gemmifera* (DC.) Zenker	Brussels Sprouts	3	2	45	1
*Brassica oleracea* L. var. g*ongylodes* L.	Turnip Kale	194	8	114	10
*Brassica oleracea* L. var. *italica* Plenck	Broccoli	20	2	20	4
*Brassica oleracea* L. var. *sabauda* L.	Savoy Cabbage	41	4	11	3
*Brassica rapa* L. ssp. *pekinensis* (Lour.) Hanelt	Chinese Cabbage	0	0	4	1
*Brassica rapa* L. ssp. *rapa* L.	Turnip	122	2	31	2
*Buxus sempervirens* L.	Common Box	4	3	21	3
*Calendula* L*. sp.*	Marigold	2	1	0	0
*Calendula officinalis* L.	Scotch Marigold	280	6	159	7
*Callistephus chinensis* (L.) Nees	China Aster	76	5	4	1
*Calystegia sepium* (L.) R. Br.	Bindweed	0	0	1	1
*Campanula carpatica* Jacq.	Carpathian Harebell	1	1	0	0
*Campanula glomerata* L.	Clustered Bellflower	8	3	0	0
*Campanula* L. sp.	Bellflower	0	0	1	1
*Campanula persicifolia* L.	Peach Leaved Bellflower	1	1	1	1
*Cannabis sativa* L.	Hemp	100	1	0	0
*Capsicum annuum* L.	Red Pepper	24	1	2	1
*Capsicum frutescens* L.	Hot Pepper	0	0	1	1
*Carum carvi* L.	Caraway	100	1	0	0
*Centaurea cyanus* L.	Cornflower	12	3	0	0
*Centaurea montana* L.	Perennial Cornflower	2	2	0	0
*Cerastium tomentosum* L.	Snow-in-Summer	3	2	4	1
*Chelidonium majus* L.	Greater Celadine	1	1	0	0
*Chrysanthemum* x *grandiflorum* (Ramat.) Kitam.	Chrysanthemum	1	1	0	0
*Cichorium endivia* L. var. *crispum* Lam.	Endive	15	1	3	1
*Cichorium endivia* L. var. *latifolium* Lam.	Escarole	105	4	6	2
*Cichorium intybus* L. var. *foliosum* Hegi	Chicory Radiccio	70	1	45	3
*Clarkia amoena* (Lehm.) A. Nelson e J.F.Macbr.	Satin Flower	10	1	0	0
*Convallaria majalis* L.	Lily-of-the-Valley	0	0	53	4
*Convolvulus tricolor* L.	Dwarf Morning Glory	3	2	0	0
*Coriandrum sativum* L.	Chinese Parsley	0	0	5	1
*Cosmos bipinnatus* Cav.	Garden Cosmos	90	4	0	0
*Cucumis sativus* L.	Cucumber	16	2	13	4
*Cucurbita maxima* Duchesne ex Lam.	Pumpkin	0	0	16	5
*Cucurbita pepo* L.	Courgette	15	4	21	8
*Cytisus scoparius* (L.) Link	Broom	0	0	1	1
*Dahlia* Cav. Cultivars	Dahlia	30	5	24	6
*Daucus carota* L. ssp. *sativus* (Hoffm.) Schübl. et G. Martens	Carrot	1746	9	1318	10
*Delphinium* L. Cultivars	Larkspur	4	3	0	0
*Deutzia* Thunb. sp.	Deutsia	1	1	0	0
*Dianthus barbatus* L.	Sweet William	186	9	52	5
*Dianthus caryophyllus* L.	Carnation	36	4	3	2
*Dianthus chinensis* L.	Annual Pink	9	2	0	0
*Dianthus deltoides* L.	Maiden Pink	0	0	1	1
*Dianthus gratianopolitanus* Vill.	Cheddar Pink	3	2	0	0
*Dianthus* L. sp.	Carnation	0	0	1	1
*Dianthus plumarius* L.	Pink	2	1	0	0
*Dianthus seguieri* Vill.		0	0	2	2
*Dicentra* Borkh. ex Bernh. Cultivars	Bleeding Heart	8	8	5	3
*Digitalis purpurea* L.	Foxglove	0	0	1	1
*Doronicum* L. sp.	Leopard's Bane	6	4	11	2
*Dorotheanthus bellidiformis* (Burm. F.) N.E.Br.	Livingstone Daisy	4	1	0	0
*Echinops bannaticus* Rochel ex Schrad.	Blue Globe Thistle	2	2	2	2
*Epilobium angustifolium* L.	Fire Weed	3	1	0	0
*Erigeron annuus* (L.) Pers.	Annual Fleabane	0	0	1	1
*Erigeron* L. Cultivars	Fleabane	1	1	0	0
*Eruca sativa* Mill.	Rocket Salad	0	0	115	6
*Eryngium planum* L.	Sea Holly	3	2	0	0
*Eschscholzia californica* Cham.	California Poppy	2	1	1	1
*Euonymus europaeus* L.	Common Spindle	0	0	1	1
*Euphorbia amygdaloides* L.	Wood Spurge	2	1	0	0
*Euphorbia helioscopia* L.	Sun Spurge	0	0	1	1
*Filipendula ulmaria* (L.) Maxim.	Meadow Sweet	0	0	1	1
*Foeniculum vulgare* Mill. ssp. *vulgare* var. a*zoricum* (Mill.) Thell.	Florence Fennel	8	1	7	1
*Forsythia* Vahl Cultivars	Forsythia	0	0	1	1
*Fragaria vesca* L. var. *vesca*	Wild Strawberry	0	0	33	3
*Fragaria vesca* L. var. h*ortensis* (Duchesne) Staudt	Strawberry	12	1	0	0
*Fragaria* x *ananassa* (Duchesne) Guédès	Garden Strawberry	324	8	182	10
*Fraxinus excelsior* L.	Common Ash	0	0	1	1
*Fritillaria imperialis* L.	Fritillary	2	1	0	0
*Galinsoga ciliata* (Raf.) S.F. Blake	Shaggy Soldier	0	0	25	1
*Galium aparine* L.	Goosegrass	0	0	30	1
*Galium odoratum* (L.) Scop	Sweet Woodruff	4	1	1	1
*Geranium* L. sp.	Crane's Bill	1	1	0	0
*Geranium robertianum* L.	Herb Robert	0	0	20	1
*Geum* L. Cultivars	Avens	9	2	5	2
*Gladiolus* L. Cultivars	Gladiolus	200	6	75	4
*Glebionis segetum* (L.) Fourr.	Corn Marigold	7	1	3	1
*Glechoma hederacea* L.	Ground Ivy	0	0	40	1
*Gynostemma pentaphyllum* (Thunb.) Makino	Jiaogulan	0	0	1	1
*Gypsophila muralis* L.	Cushion Baby's Breath	0	0	1	1
*Gypsophila paniculata* L.	Baby's Breath	2	2	2	1
*Hebe* Comm. Ex Juss. Cultivars	Hedge Veronica	0	0	1	1
*Helianthus annuus* L.	Common Sunflower	39	2	25	3
*Helianthus pauciflorus* Nutt.	Pauciflorus	15	1	27	1
*Helianthus tuberosus* L.	Jerusalem Artichoke	0	0	20	3
*Heliopsis helianthoides* (L.) Sweet var. s*cabra* (Dunal) Fernald	Ox Eye	1	1	6	2
*Hemerocallis fulva* (L.) L.	Orange Daylily	0	0	10	4
*Hemerocallis* L. Cultivars	Daylily	5	3	14	5
*Heracleum sphondylium* L.	Hogweed	0	0	1	1
*Humulus lupulus* L.	Common Hop	0	0	1	1
*Hydrangea* L. Cultivars	Hydrangea	1	1	5	3
*Hypericum perforatum* L.	St. John's Wort	2	2	8	2
*Hyssopus officinalis* L.	Hyssop	1	1	2	2
*Iberis amara* L.	Wild Candytuft	3	1	0	0
*Iris pumila* L. Cultivars	Dwarf Flag	10	1	31	1
*Iris sibirica* L. Cultivars	Siberian Iris	2	2	0	0
*Iris* x *germanica* L. Cultivars	Common Iris	27	6	42	4
*Juniperus communis* L.	Common Juniper	1	1	0	0
*Laburnum anagyroides* Medik.	Common Laburnum	1	1	0	0
*Lactuca sativa* L. var. c*apitata* L.	Cabbage Lettuce	437	16	425	14
*Lactuca sativa* L. var. *crispa* L.	Leaf Lettuce	90	6	26	9
*Lamium album* L.	White Dead Nettle	0	0	30	1
*Lamium purpureum* L.	Purple Archangel	0	0	13	1
*Lavandula angustifolia* Mill.	English Lavender	4	3	5	3
*Leontopodium nivale* (Ten.) A. Huet ex Hand.-Mazz.	Edelweiss	2	2	11	2
*Leonurus cardiaca* L.	Motherwort	0	0	5	1
*Lepidium sativum* L.	Garden Cress	18	4	100	1
*Leucanthemum heterophyllum* (Willd.) DC.	Oxeye Daisy	0	0	1	1
*Leucanthemum ircutianum* DC.	Oxeye Daisy	0	0	2	1
*Leucanthemum* Mill. sp.	Oxeye Daisy	8	4	34	3
*Levisticum officinale* W.D.J. Koch	Lovage	9	9	11	11
*Liatris spicata* (L.) Willd.	Button Snakeroot	1	1	0	0
*Lilium bulbiferum* L.	Fire Lily	6	3	8	2
*Lilium candidum* L.	Madonna Lily	0	0	9	1
*Lilium* L. Cultivars	Lily	20	6	45	6
*Lilium lancifolium* Thunb.	Tiger Lily	0	0	10	1
*Lilium martagon* L.	Martagon Lily	1	1	0	0
*Limonium* Mill. sp.	Sea Lavender	20	3	2	1
*Linaria maroccana* Hook.	Baby Snapdragon	4	1	0	0
*Linum grandiflorum* Desf.	Red Flax	4	1	60	1
*Linum usitatissimum* L.	Common Flax	200	1	0	0
*Lobularia maritima* (L.) Desv.	Sweet Alsion	5	1	0	0
*Lonicera caprifolium* L. sp.	Honeysuckle	0	0	1	1
*Lunaria annua* L.	Annual Honesty	1	1	0	0
*Lupinus* L. Cultivars	Garden Lupin	13	4	8	4
*Lycium barbarum* L.	Box Thorn	0	0	2	1
*Lycopersicon esculentum* Mill.	Tomato	9	2	21	4
*Lysimachia punctata* L.	Dotted Loosestrife	19	5	11	6
*Mahonia aquifolium* (Pursh) Nutt.	Oregon Grape	1	1	0	0
*Malus domestica* Borkh.	Apple	2	1	5	2
*Malva neglecta* Wallr.	Common Mallow	4	1	2	1
*Malva sylvestris* L. ssp. *mauritiana* (L.) Boiss. Ex Cout.	Mallow	2	1	0	0
*Malva sylvestri*s L. ssp. *sylvestris*	Blue Mallow	0	0	1	1
*Malva verticillata* L.	Curled Mallow	0	0	13	1
*Marrubium vulgare* L.	Common Horehound	2	1	0	0
*Matricaria discoidea* DC.	False Chamomile	0	0	23	1
*Matricaria recutita* L.	Chamomile	82	8	83	6
*Mauranthemum paludosum* (Poir.) Vogt et Oberpr.		2	1	0	0
*Medicago lupulina* L.	Black Medick	0	0	3	1
*Melissa officinalis* L.	Lemon Balm	5	3	11	5
*Mentha* L. sp.	Mint	10	3	5	1
*Mentha longifolia* (L.) L.	Horse Mint	1	1	0	0
*Mentha spicata* L.	Spearmint	0	0	1	1
*Mentha suaveolens* Ehrh.	Apple Mint	3	2	46	4
*Mentha* x *piperita* L.	Peppermint	6	2	14	4
*Mirabilis jalapa* L.	Four O'Clock Plant	5	1	0	0
*Monarda* L. Cultivars	Beebalm	3	1	3	3
*Myosotis* L. sp.	Forget-me-not	6	3	1	1
*Nepeta cataria* L.	Cat Mint	5	4	7	5
*Ocimum basilicum* L.	Basil	0	0	33	5
*Origanum majorana* L.	Sweet Marjoram	11	4	7	5
*Origanum vulgare* L.	Oregano	3	1	9	5
*Osteospermum* L. Cultivars	African Daisy	0	0	9	1
*Paeonia lactiflora* Pall. Cultivars	Common Garden Peony	34	11	27	8
*Paeonia officinalis* L.	Common Peony	16	8	3	3
*Papaver nudicaule* L.	Iceland Poppy	2	1	9	1
*Papaver orientale* L.	Oriental Poppy	1	1	3	2
*Papaver somniferum* L. ssp. *setigerum* (DC.) Corb.	Poppy	0	0	2	1
*Papaver somniferum* L. ssp. *somniferum*	Opium Poppy	1	1	0	0
*Persicaria lapathifolia* (L.) Delarbre	Pale Persicaria	0	0	1	1
*Petroselinum crispum* (Mill.) Fuss	Parsley	44	12	496	11
*Phaseolus vulgaris* L. var. n*anus* (L.) G. Martens	Dwarf Bean	457	7	460	6
*Phaseolus vulgaris* L. var. *vulgaris*	French Bean	20	1	38	3
*Philadelphus* L. Cultivars	Mock Orange	1	1	1	1
*Phlox paniculata* L.	Garden Phlox	20	7	45	10
*Phlox subulata* L.	Moss Phlox	7	2	0	0
*Physalis alkekengi* L.	Chinese Lantern Plant	5	1	0	0
*Physostegia virginiana* (L.) Benth.	Obedient Plant	6	2	1	1
*Pinus cembra* L.	Arolla Pine	2	1	2	1
*Pisum sativum* L. ssp. *sativum*	Garden Pea	746	7	65	2
*Plantago lanceolata* L.	English Plantain	1	1	9	1
*Plantago major* L.	Common Plantain	0	0	25	1
*Polemonium* L. Cultivars	Jacob's Ladder	0	0	1	1
*Potentilla fruticosa* L.		1	1	0	0
*Prunus armeniaca* L.	Apricot	0	0	1	1
*Prunus avium* (L.) L.	Gean	0	0	2	2
*Prunus domestica* L.	Plum	1	1	8	1
*Prunus triloba* Lindl.	Flowering Almond	0	0	1	1
*Psyllium* Mill. sp.	Plantain	3	1	0	0
*Pulmonaria officinalis* L.	Lungwort	0	0	1	1
*Pyrus communis* L.	Common Pear	1	1	1	1
*Raphanus sativus* L. var. ni*ger* (Mill.) J.Kern.	Oriental Radish	30	1	0	0
*Raphanus sativus* L. var. *sativus*	Small Radish	310	9	231	7
*Rheum rhabarbarum* L.	Garden Rhubarb	10	7	5	5
*Ribes nigrum* L.	Blackcurrant	6	2	28	6
*Ribes rubrum* L.	Red Currant; White Currant	29	4	45	8
*Ribes uva-crispa* L.	Gooseberry	0	0	3	2
*Ribes* x *nidigrolaria* Rud. Bauer et A. Bauer		1	1	9	1
*Rosa* L. Cultivars	Rose	20	10	21	9
*Rosa* L. sp.	Rose	0	0	2	2
*Rosa* x *alba* L.	White Rose	1	1	0	0
*Rosa* x *centifolia* L.	Rose	4	3	3	2
*Rosmarinus officinalis* L.	Rosemary	1	1	6	5
*Rubus idaeus* L.	Raspberry	9	5	42	5
*Rubus* L. sect. *Rubus*		0	0	1	1
*Rudbeckia hirta* L.	Black-Eyed Susan	0	0	50	1
*Rudbeckia laciniata* L.	Cutleaf Coneflower	1	1	0	0
*Rumex obtusifolius* L.	Bitter Dock	0	0	8	1
*Ruta graveolens* L.	Rue	0	0	2	2
*Sagina subulata* (Sm.) C. Presl	Pearlwort	5	1	0	0
*Salvia officinalis* L.	Common Sage	6	6	8	8
*Sambucus nigra* L.	Common Elder	0	0	1	1
*Sanguisorba minor* Scop.	Small Burnet	2	1	0	0
*Santolina chamaecyparissus* L.	Lavender Cotton	0	0	1	1
*Satureja hortensis* L.	Summer Savory	10	1	2	2
*Satureja montana* L.	Winter Savory	1	1	1	1
Saxifraga L. Cultivars	Saxifrage	4	2	0	0
*Scorzonera hispanica* L.	Black Salsify	8	1	0	0
*Sedum acre* L.	Wall Pepper	0	0	1	1
*Sedum cauticola* Praeger	Stonecrop	2	1	0	0
*Sedum hispanicum* L.	Stonecrop	9	3	0	0
*Sedum* L. sp.	Stonecrop	0	0	1	1
*Sedum spurium* M. Bieb	Two Row Stonecrop	3	2	4	2
*Sedum telephium* L.	Stonecrop	3	2	2	2
*Silene chalcedonica* (L.) E.H.L. Krause	Maltese Cross	0	0	1	1
*Silene coronaria* (L.) Clairv.	Crown Pink	0	0	1	1
*Silene latifolia* Poir.	White Campion	5	1	0	0
*Silybum marianum* (L.) Gaertn.	Our Lady's Thistle	0	0	1	1
*Solanum tuberosum* L.	Potato	590	4	220	5
*Solidago canadensis* L.	Canada Goldenrod	3	2	5	1
*Sorbus aucuparia* L.	Mountain Ash	1	1	1	1
*Spinacia oleracea* L.	Spinach	16	3	38	2
*Spiraea japonica* L.	Japanese Meadowsweet	1	1	3	2
*Spiraea* L. sp	Bridewort	1	1	1	1
*Stellaria media* (L.) Vill.	Common Chickweed	0	0	60	1
*Symphoricarpos albus* (L.) S.F. Blake	Snowberry	4	2	5	2
*Symphytum asperum* Lepech.	Rough Comfrey	0	0	1	1
*Symphytum officinale* L.	Common Comfrey	2	1	13	6
*Syringa vulgaris* L.	Common Lilac	6	2	4	3
*Tagetes* L. sp.	Marigold	94	5	13	4
*Tanacetum coccineum* (Willd.) Grierson	Painted Daisy	2	2	0	0
*Tanacetum parthenium* (L.) Sch. Bip.	Feverfew	1	1	4	3
*Tanacetum vulgare* L.	Tansy	1	1	2	2
*Taraxacum* sect. *Ruderalia* Kirschner	Dandelion	0	0	20	1
*Thuja occidentalis* L.	Red Cedar	0	0	4	2
*Thymus pulegioides* L.	Thyme	0	0	3	2
*Thymus vulgaris* L.	Common Thyme	4	2	3	3
*Thymus* x *citriodorus* (Pers.) Schreb.	Lemon Thyme	1	1	1	1
*Tilia cordata* Mill.	Little Leaf Linden	1	1	0	0
*Tilia platyphyllos* Scop.	Large Leaved Lime	1	1	0	0
*Tradescantia* x *andersoniana* W. Ludw. et Rohweder	White Spiderwort	1	1	0	0
*Trifolium pratense* L.	Red Clover	0	0	30	1
*Trigonella caerulea* (L.) Ser.	Fenugreek	21	2	3	3
*Tropaeolum majus* L.	Nasturtium	41	5	28	3
*Tussilago farfara* L.	Coltsfoot	0	0	5	1
*Urtica dioica* L.	Stinging Nettle	10	1	43	4
*Vaccinium corymbosum* L.	High Bush Blueberry	4	1	4	2
*Valeriana officinalis* L.	Common Valerian	3	2	1	1
*Valerianella locusta* (L.) Laterr.	Cornsalad	9	1	21	1
*Verbascum densiflorum* Bertol.	Large-flowered Mullein	0	0	6	3
*Verbascum olympicum* Boiss.	Olympic Mullein	2	1	0	0
*Verbena officinalis* L.	Turkey Grass	2	1	0	0
*Viburnum lantana* L.	Wayfaring Tree	0	0	1	1
*Viburnum opulus* L.	European Cranberrybush	0	0	2	2
*Vicia faba* L.	Broad Bean	150	1	0	0
*Vicia sepium* L.	Bush Vetch	0	0	5	1
*Vinca minor* L.	Smaller Periwinkle	0	0	10	5
*Viola arvensis* Murray	European Field Pansy	0	0	1	1
*Viola* x *wittrockiana* Gams ex Kappert	Garden Pansy	24	4	15	2
*Vitis vinifera* L. ssp. *vinifera*	Common Grape Vine	0	0	5	2
*Weigelia* Thunb. Cultivars	Weigela	0	0	1	1
*Xerochrysum bracteatum* (Vent.) Tzvelev	Straw Daisy	20	2	5	2
*Zinnia elegans* Jacq.	Youh-and-Old-Age	87	4	8	2

**Table 3:**
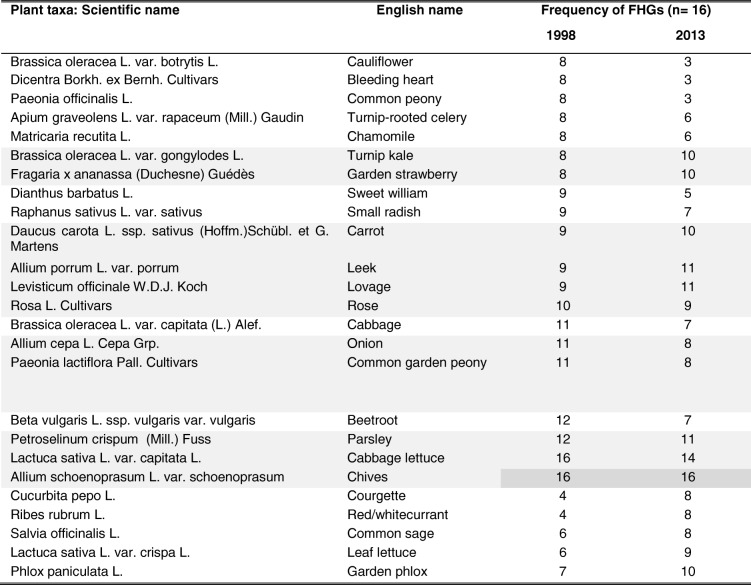
Plant taxa found in > 50% of the studied home gardens in at least one of the study years of 1998 or 2013 (*n* = 16 each year). Grey: > 50% in both years

**Fig. 3:**
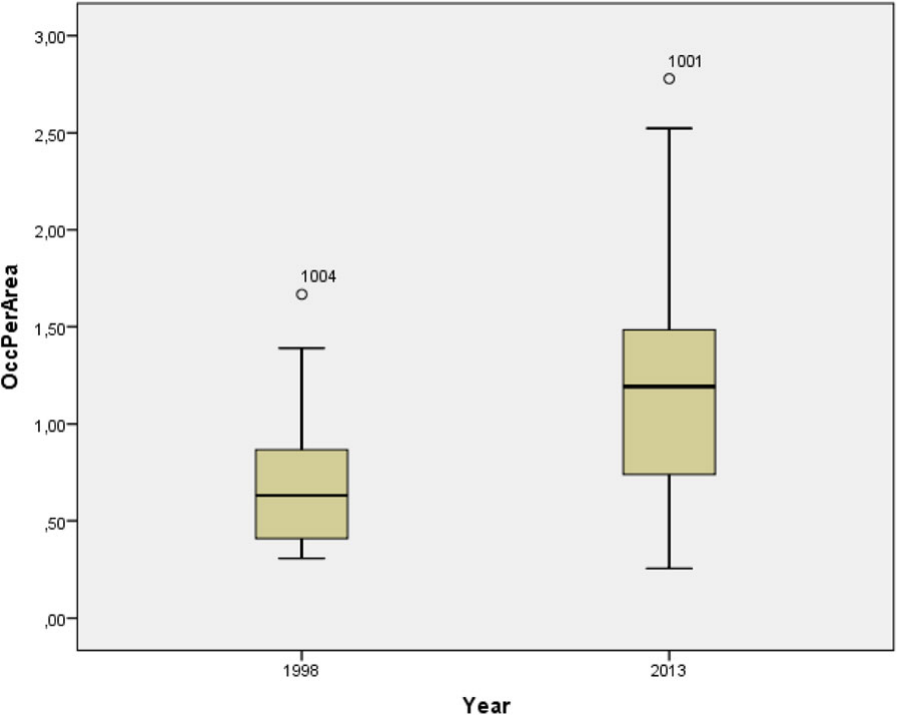
FHG-specific diversity (occurrence/m^2^) for *n* = 16 in 1998 and the same *n* = 16 in 2013, shown as a box plot with median

FHG diversity (occ./m^2^) increased significantly (p_t-test paired_ = 0.021) between 1998 (arithmetic mean 0.7) and 2013 (arithmetic mean 1.2) (Fig. 3, Table 1). The highest FHG diversity in 2013 was found in FHG_1001, FHG_1003 and FHG_1004. Two of these gardens (FHG_1001, FHG_1003) had been considerably reduced in size and, according to the gardeners, a reduction was the only way the FHG could be maintained given the time available for gardening—either by maintaining or even increasing the occurrence of plant taxa. Both gardeners expressed unhappiness with gardening. The gardener of FHG_1003 mentioned that it would be possible to continue farming or being a farmer without having a FHG. The gardener of FHG_1004 was one of the three “keen gardeners”.

**Fig. 4:**
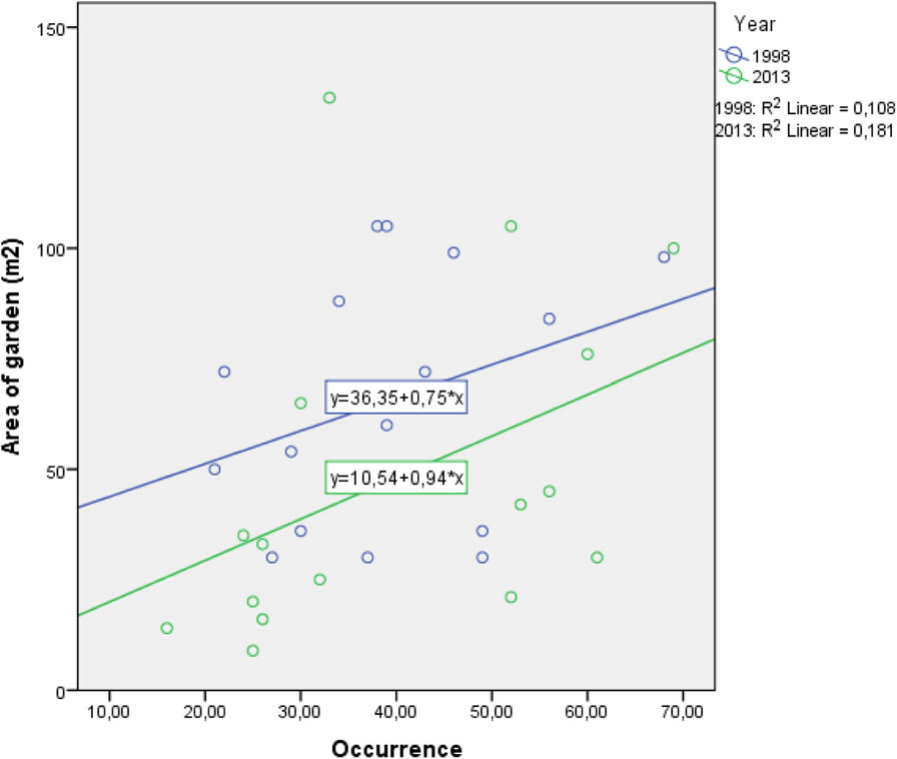
Scatter plot for garden size and occurrence for *n* = 16 in 1998 and the same *n* = 16 in 2013

The correlation between FHG size and occurrence was not significant for 1998 (*p*_PEARSON_ = 0.214; correlation coefficient 0.329) or 2013 (*p*_PEARSON_ = 0.101; correlation coefficient = 0.425), i.e. small gardens might host many different plant taxa or large gardens might have fewer plant taxa (Fig. 4).

FHGs may be characterised as being primarily managed for ornamental and food uses, as the absolute number of plant taxa with ornamental use prevailed in both years (116 plant taxa in 1998 and 155 in 2013), followed by plant taxa used as food (79 plant taxa in 1998 and 122 in 2013). Of the eleven plant taxa occurring in ≥ 50% of the FHGs in both the surveyed years and contributing to the similarity between the years, food function was represented by nine plant taxa. However, as ornamental and food uses were never the only function mentioned by the gardeners (both in 1998 and 2013), FHGs could therefore be perceived overall as multifunctional systems.

In FHGs in Sillian, 26 plant taxa in 1998 and 117 in 2013 were categorised in more than one functional group, e.g. nine plant taxa in 1998 and 45 in 2013 were mentioned both for medicine and food use (or food subcategories), such as:

- Medicinal and food/spice (e.g. *Salvia officinalis* L., *Borago officinalis* L.);
- Medicinal and food/vegetable (e.g. *Aegopodium podagraria* L., *Urtica dioica* L.);
- Medicinal and eaten raw as food/salad (e.g. *Rumex obtusifolius* L., *Galinsoga ciliata* (Raf.) S.F. Blake);
- Medicinal and food/beverage (e.g. *Nepeta cataria* L., *Mentha x piperita* L.).

The variation in the different use categories expressed in the occurrence between the years was not significantly different, except for the increase in the occurrence of plant taxa used as food (*p*_*t* test_ = 0.009, significant) and the food subcategory spice (*p*_*t* test_ = 0.056, tendency) (Table 1).

#### Abundance (frequency of individuals)

**Fig. 5:**
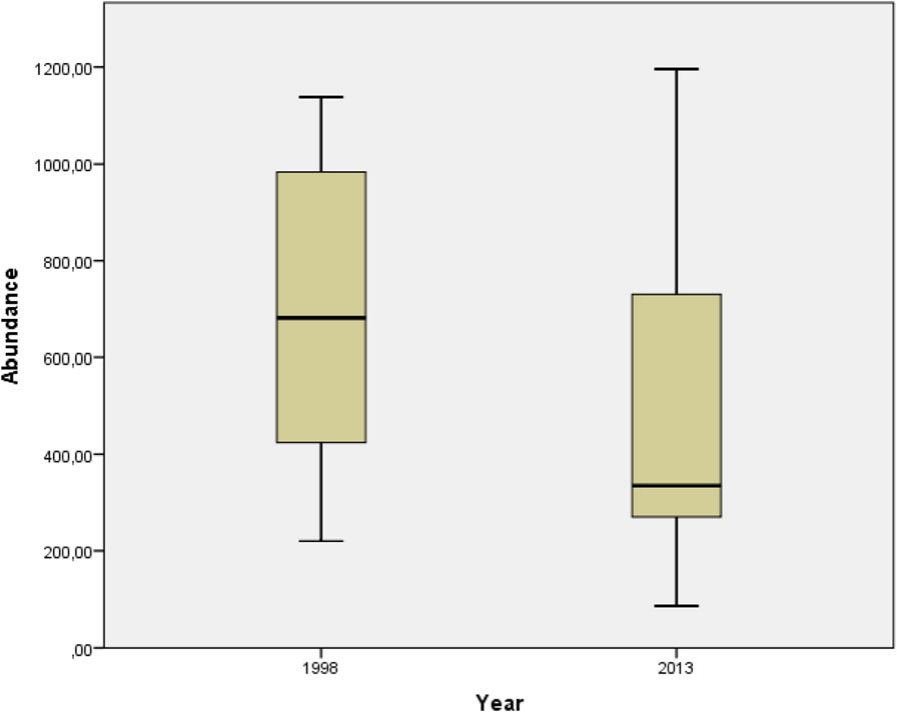
Abundance (frequency of individuals of plant taxa) per FHG for *n* = 16 in 1998 and the same *n* = 16 in 2013, shown as a box plot with median

The abundance of all FHGS was 11,068 individuals in 1998 and 7794 individuals in 2013. The mean abundance of individuals for all plant taxa showed a significant decrease for the mean of individual plants per garden between the years (1998, 691; 2013, 487; *p*_*t* test paired_: 0.027) (Table 1). In 2013, an increase in variability (e.g. expressed in the standard deviation; Table 1) compared to 1998 could be seen, i.e. FHGs with a higher maximum and FHGs with a lower minimum number of individuals per FHG could to be observed compared to 1998 (Fig. 5).

The two FHGs with the greatest abundance in 2013 could be characterised as gardens, with gardeners using spontaneously growing plant taxa (FHG_1015; e.g. ground elder; *Aegopodium podagraria* L., 100 individuals or *Glechoma hederacea* L., 40 individuals), or growing spice plant taxa with a high abundance (FHG_1013; e.g. *Petroselinum crispum* (Mill.) Fuss, 240 individuals).

There were no significant differences in relative abundance between the years (*p*_*t* test paired_ = 1.000), with FHGs holding between 1% (FHG_1001) and 15% (FHG_1015) of the total abundance in 2013. FHG abundance showed no difference between the years (*p*_*t* test paired_ = 0.700). FHGs had between three and 19 plant individuals per m^2^ (Table 1).

**Fig. 6:**
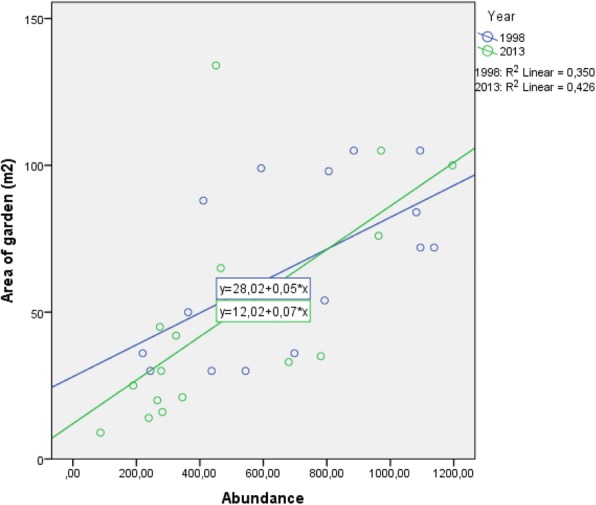
Scatter plot (including correlation) for abundance and size of garden for 1998 and 2013 (*n* = 16).

A significant correlation between FHG size and abundance could be observed for 1998 (*p*_PEARSON_ = 0.016; correlation coefficient 0.591) and 2013 (*p*_PEARSON_ = 0.006; correlation coefficient = 0.653) (Fig. 6).

The variation in the different use categories expressed in abundance between the years was not significantly different, except for the decrease in the abundance of plant taxa used as food (*p*_*t* test paired_ = 0.047). In spite of the significant decrease in the abundance of food on average, for certain plant taxa their abundance increased (Table 1), e.g. the abundance of *Petroselinum crispum* (Mill.) Fuss.

### Most recent changes in spatial configuration in 2017

**Fig. 7:**
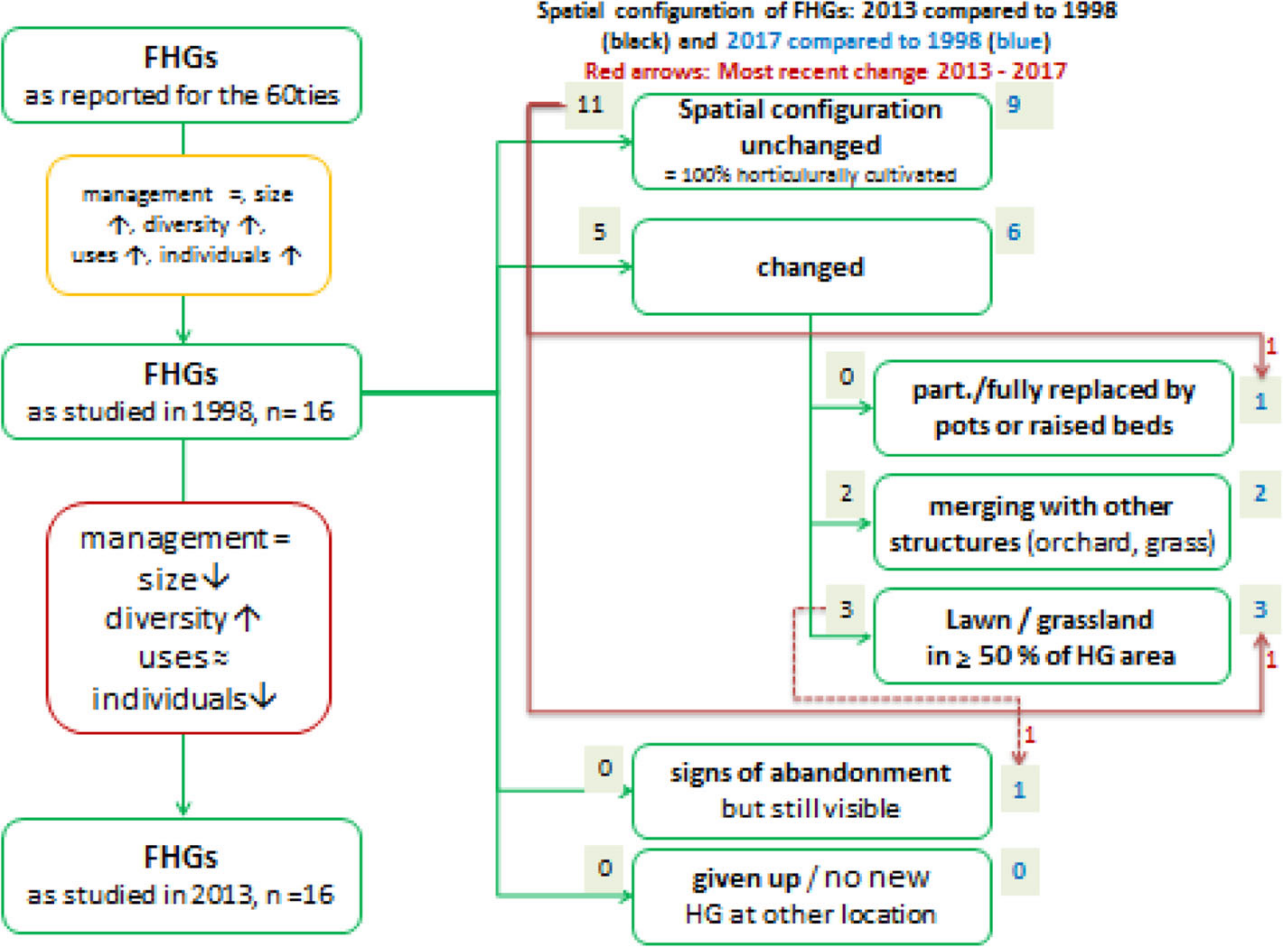
Changes observed in FHGs in Sillian (Eastern Tyrol). Yellow box: changes between the 1960s and 1998 as published; red box: changes as presented in this paper 1998 > 2013 (= situation maintained; ↑ increased; ↓ decreased; ≈ increased/decreased/similar depending on use category); black or blue figures in grey boxes indicate frequency of FHGs for the respective category—only related to changes in spatial configuration; *n* = 16

Between 2013 and 2017 three FHGs showed very recent changes in their spatial configuration, while 13 remained as observed in 2013 (Fig. 7).

The traditional spatial configuration of FHG_1009 and FHG_1020 changed between 2013 and 2017 by (i) converting ≥ 50% of the garden into grassland or (ii) replacing the FHG with one raised bed. Horticultural activities could clearly be observed.

Already in 2013 in FHG_1003 ≥ 50% of the area under cultivation had been converted into lawn. In 2017, only some perennial shrubs and flowering plants served as a reminder of the FHG. Between 2013 and 2017 the area had been fully converted into pastureland. Two sides of the all-surrounding fence had been removed and no horticultural activity could be observed.

## Discussion

Between 1998 and 2013, incipient change was apparent in the spatial configuration of the 16 studied FHGs in Sillian, with this continuing trend also visible in 2017.

While the boundaries of the FHG plots were accurately defined until 1998 and contributed to a homogeneous picture of these environments [18], some FHGs had now expanded into other functional structures of the homestead (e.g. orchards or grassland), including recreational areas and lawn with more open boundaries (semi-fenced or not fenced) or new structural elements (e.g. raised beds). A common attribute of tropical home gardens is the diversity in the spatial configurations of cultivated spaces [24, 44–47] and the gradual transition of home gardens to surrounding areas [48], contributing to the heterogeneity of these agroecosystems and making the definition of boundaries unfeasible [44]. Indeterminate boundaries, which make the measurement of size subjective and inaccurate, e.g. in Benin [44], were not observed in 1998, but were observed in 2013 and 2017. For temperate FHGs, we have not found scientific evidence discussing fences or boundaries. We hypothesise that presence/absence of fences might be an indicator of change for the FHGs studied.

The decrease in the cultivated area of FHGs in Sillian may be linked to challenges in the management of the FHGs, as indicated by the gardeners. Strategies for avoiding high labour demand included reducing the size of the FHG and growing plant taxa such as redcurrant that require less time-consuming maintenance and cover more space per individual, maintaining or even expanding the garden size. Therefore raised beds also appeared to be a strategy applied by the gardeners, as they demand less labour and are easier to manage, especially by the older generation.

It can be argued that management factors, e.g. time to invest in gardening, should be more strongly considered as predictors of structural conversion or abandonment. Furthermore, other household features have to be taken into account in future evaluations, e.g. whether better-off households (defined by wealth in land and kinship affiliation [19]) have more time to invest in gardening and therefore tend to have greater agrobiodiversity, as observed in Peru for example [19].

Although some gardening activities in East Tyrolean FHGs in 1998 were shared between different household members (e.g. digging by men), women were mainly responsible for gardening (100% in 1998 in Sillian FHGs), which was also reported for the 1960s [13]. This differs from research into FHGs on the Iberian Peninsula, for example, which suggests that men have main responsibility for gardening activities in 52% of the households or that it is shared by family members in 21% of the households [42]. In 2013 in Sillian FHGs, the first steps towards gardening being shared more between family members or becoming a man’s domain might indicate a transition in the cultural roles expected of household members in the management of FHGs.

The incipient change in spatial configuration and the heterogeneous nature of FHGs may be seen as an ongoing expression of individualisation. It can be argued that farmers’ home gardening might be undergoing a process of reconsideration of culturally-rooted forms of home gardening. In future home gardens, seen as a marker of cultural identity in other regions in Europe [49], may become more differentiated, in line with farmers’ perceptions of what a FHG means for them individually.

No difference was observed between the mean occurrences of plant taxa between the years 1998 and 2013. Nevertheless, in 2013, the gardeners had increased the absolute number of all plant taxa in all 16 FHGs and FHG-specific diversity (occ./m^2^) had increased. In neither of the periods surveyed was diversity related to the size of the garden, as observed in tropical home gardens for example [19, 31], but unlike home gardens in Nepal [50], where size and species richness were positively correlated, or Vietnam [51] where smaller FHGs showed greater diversity. While households with an interest in plant taxa tend to have more diverse gardens, e.g. in Peru [19], in Sillian, more diverse gardens were not clearly related to the enthusiasm for gardening. One of the highly diverse gardens in the village was abandoned in 2017.

Trends in home garden dynamics in other regions are showing a gradual decrease in diversity and structural simplification as a result of intensification of crop production [24, 52]. FHGs are therefore described as being under threat of conversion to mono-cropping land use systems [28, 53].

FHGs in Sillian face a “different kind of threat”, i.e. a continuous process of decline in the cultivated area, radical structural change (e.g. conversion of the FHG to one raised bed, as observed in one FHG in 2017) or the complete abandonment of the horticulturally managed system and conversion to another land-use system such as pasture (observed in one FHG in 2017). In FHGs in Germany, Schulmeyer-Torres [22] identifies the 1990s as a period in which structural changes to a high proportion of lawn and coniferous plant taxa might have taken place. Such a change can be confirmed for 2013 onwards in Sillian.

The flora in the studied gardens was highly variable in both 1998 and 2013, suggesting the heterogeneous nature of FHGs, also observed by [53] and indicated by [19] as substantial differences in garden composition and plant diversity between households. Although no significant difference in the number of occurrence of plant taxa (per garden) between the years was observed, an increase in the variability of the occurrence of plant taxa, an increase in the absolute number of plant taxa that entered the FHGs than left them, and fewer plant taxa that contributed to the similarity in 2013 showed that the differences between FHGs increased over the years. This increase in variability might be understood as a flora-specific indicator of individualisation of the composition of the FHGs based on the gardeners’ differing preferences.

The multifunctional characteristics of FHGs found in Eastern Tyrol in 1998 could also be confirmed for 2013, similar to tropical home gardens [3]. In comparison, e.g. on the Iberian Peninsula, a specialised role of home gardens for food production with a low frequency of nonedible taxa was found [54].

The main function of the historic FHGs until the 1960s was to provide the household with edible plant taxa for use as seasoning, besides their medicinal function, with both functions continuing to be important in 1998. In 2013, human medicinal uses continued to play a steady role, whereas food uses showed an increase of occurrence of plant taxa for 2013 and a decrease in the abundance of individuals cultivated.

These findings may be interpreted as showing that it is not large quantities (abundance) that are important, but the taste and health of garden products of specific taxa (occurrence) for the diet of the gardeners. This is also obvious in the increase of the occurrence of plant taxa used as spices (spice being a subcategory of food).

In several studies, spices are not just valued as seasoning, but seen as plants with medicinal and/or therapeutic potential [55] and are therefore the subject of experimental research on their health benefits, based on the knowledge of the chemistry and pharmacology of their active principles [56]. Viewing food use as nutritional or medicinal is often only a matter of definition and thus difficult to assess [57]. Since half of the food plants in Northern Spain [15] or 60% in Lucca, Italy [58] for example have medicinal uses, there are differing degrees of correlations along the food-medicine continuum [57]. The emic perception of this continuum might be of interest for further evaluation in East Tyrolean FHGs.

The use of plant taxa for healthy food and medicinal purposes was consistent with the respondents who called themselves “keen” gardeners making management decisions linked to use categories. The knowledge associated with “healthy” plants grown in FHGs in Sillian might be of conservation significance, as stated by Huai et al. [59]. A prime requirement for success in conservation is the presence of local people who are knowledgably about their local natural world, e.g. medicinal plants. A link between use categories and agrobiodiversity maintenance—the more functions there are, the better it is for agrobiodiversity, as observed elsewhere [19, 60, 61]—might also be true for FHGs in Sillian, which still show high plant diversity and multifunctional characteristics.

Finally, the exchange of seeds and planting material is recognised as an important determinant of diversity, with implications especially for in situ conservation of varietal diversity [19]. In 1998, interviews still recorded the regional sources for seeds and planting material [20]. This regional economy might be affected by the increased expression of individualisation in the garden system of the 16 Sillian FHGs, particularly in a system where markets for seeds and planting material are omnipresent. Access to and the origin and dynamic of garden planting material were not investigated in 2013. This issue merits closer attention in studies of agrobiodiversity [19, 50] and should be taken into account in future research. Future studies should include the importance of assessing diversity through more extensive sampling and giving more extensive consideration to the underlying socioecological and sociocultural drivers that lead to the fragmentation, conversion or abandonment of FHGs.

## Conclusions

The diachronic perspective of this study provides a first understanding of the ongoing conversion underway in FHGs at different points in time (1998, 2013, 2017 of the same 16 FHGs), including changes in the 1960s for FHGs in the study area. In Sillian, FHGs are by no way distinct or static agroecological units, but are dynamic in their appearance, composition and function. FHGs in Sillian show a trend towards becoming more individual, i.e. conversion from being a product of a homogenous local cultural script of the community into an area where gardeners define more individually the role that FHGs are expected to play for them or their family. As an FHG may no longer consist of a single fenced plot, but instead cover various locations or growing sites, a careful redefinition should be made of the system boundary comprised by a “FHG” in Eastern Tyrol. This observation might also be of relevance in the design of further studies in other regions.

## Abbreviations

FHG: Farmer’s homegarden
FHGs: Farmers’ homegardens

## References

[1] Landauer K, Brazil M (1990). Tropical home gardens.

[2] Watson JW, Eyzaguirre PB (2002). Home gardens and in situ conservation of plant genetic resources in farming systems. Proceedings of the Second International Home Gardens Workshop; Witzenhausen, Germany.

[3] Kumar BM, Nair PKR (2004). The enigma of tropical homegardens. Agrofor Syst.

[4] Vogl CR, Vogl-Lukasser B, Caballero J, Stepp JR, Wyndham FS, Zarger RK (2002). Homegardens of Maya migrants in the district of Palenque (Chiapas/Mexico): implications for sustainable rural development. Ethnobiology and biocultural diversity.

[5] Neulinger K, Vogl CR, Alayón-Gamboa JA (2013). Plant species and their uses in homegardens of migrant maya and mestizo smallholder farmers in Calakmul, Campeche, Mexico. J Ethnobiol.

[6] Calvet-Mir L, March H, Corbacho-Monné D, Gómez-Baggethun E, Reyes-García V. Home garden ecosystem services valuation through a gender lens: a case study in the Catalan Pyrenees. Sustainability (Switzerland). 2016;8.

[7] Calvet-Mir L, Riu-Bosoms C, González-Puente M, Ruiz-Mallén I, Reyes-García V, Molina JL (2016). The transmission of home garden knowledge: safeguarding biocultural diversity and enhancing social–ecological resilience. Soc Nat Resour.

[8] Agelet A, Bonet MA, Vallès J (2000). Homegardens and their role as a main source of medicinal plants in montain regions of Catalonia (Iberian Peninsula). Econ Bot.

[9] Plieninger T, Höchtl F, Spek T (2006). Traditional land-use and nature conservation in European rural landscapes. Environ Sci Policy.

[10] Rachewilz D (1983). Brot im Südlichen Tirol.

[11] Ermann U, Langthaler E, Penker M, Schermer M (2017). Agro-food studies: Eine Einführung.

[12] Wolf R (2016). Die Alpenkonvention. Natur und Recht.

[13] Vogl-Lukasser B. Studien zur funktionalen Bedeutung bäuerlicher Hausgärten in Osttirol basierend auf Artenzusammensetzung und ethnobotanischen Analysen. In: Dissertation: University of Vienna, Institute for Ecology and Conservation Biology; 2000.

[14] Christanell A, Vogl-Lukasser B, Vogl CR, Gütler M (2010). The cultural significance of wildgathered plant species in Kartitsch (Eastern Tyrol, Austria) and the influence of socioeconomic changes on local gathering practices. Ethnobotany New Europe People Health Wild Plant Resour.

[15] Pardo de Santayana M, San Miguel E, Morales R, Pieroni A, Price LL (2006). Digestive beverages as a medicinal food in a cattle-farming community in northern Spain (Campoo, Cantabria). Eating and healing - traditional food as medicine.

[16] Vogl CR, Vogl-Lukasser B, Walkenhorst M (2016). Local knowledge held by farmers in Eastern Tyrol (Austria) about the use of plants to maintain and improve animal health and welfare. J Ethnobiol Ethnomed.

[17] Vogl-Lukasser B, Vogl CR (2004). Ethnobotanical research in homegardens of small farmers in the alpine region of Osttirol (Austria): an example for bridges built and building bridges. Ethnobot Res Appl.

[18] Vogl-Lukasser B, Vogl CR. Ethnobotanical research in homegardens of small farmers in the alpine region of Osttirol (Austria): photo essay. Ethnobot Res Appl. 2005:79–97.

[19] Coomes OT, Ban N (2004). Cultivated plant species diversity in home gardens of an amazonian peasant village in northeastern Peru. Econ Bot.

[20] Vogl CR, Vogl-Lukasser B (2003). Tradition, dynamics and sustainability of plant species composition and management in homegardens on organic and non-organic small scale farms in alpine Eastern Tyrol, Austria. Biol Agric Horticulture.

[21] Van Der Stege C, Vogl-Lukasser B, Vogl CR (2010). The role of homegardens in strengthening social—ecological resilience: case studies from Cuba and Austria. Resilience and the cultural landscape: understanding and managing change in human-shaped environments.

[22] Schulmeyer-Torres D (1994). Bauerngärten: Historische Entwicklung und Charakterisierung des aktuellen Artenbestandes der ländlichen Hausgärten in West-Miteleuropa anhand ökologischer und historisch-geographischer Merkmale - Ein Beitrag zur Erforschung der Überreste des Bauerngartens.

[23] Michon G, Mary F (1994). Conversion of traditional village gardens and new economic strategies of rural households in the area of Bogor, Indonesia. Agrofor Syst.

[24] Abebe T, Wiersum KF, Bongers F (2010). Spatial and temporal variation in crop diversity in agroforestry homegardens of southern Ethiopia. Agrofor Syst.

[25] Peyre A, Guidal A, Wiersum KF, Bongers F (2006). Dynamics of homegarden structure and function in Kerala, India. Agrofor Syst.

[26] Chandrashekara UM, Baiju EC (2010). Changing pattern of species composition and species utilization in homegardens of Kerala, India. Trop Ecol.

[27] Woldeyes F, Asfaw Z, Demissew S, Roussel B (2016). Homegardens (Aal-oos-gad) of the basket people of southwestern Ethiopia: sustainable agro-ecosystems characterizing a traditional landscape. Ethnobot Res Appl.

[28] Gebrehiwot M, Elbakidze M, Lidestav G, Sandewall M, Angelstam P, Kassa H (2016). From self-subsistence farm production to khat: driving forces of change in Ethiopian agroforestry homegardens. Environ Conserv.

[29] Galluzzi G, Eyzaguirre P, Negri V (2010). Home gardens: neglected hotspots of agro-biodiversity and cultural diversity. Biodivers Conserv.

[30] Ninez VK (1985). Household food production - comparative perspectives.

[31] Blanckaert I, Swennen RL, Paredes Flores M, Rosas López R, Lira Saade R (2004). Floristic composition, plant uses and management practices in homegardens of San Rafael Coxcatlán, Valley of Tehuacán-Cuicatlán, Mexico. J Arid Environ.

[32] Staller M, Lehrerverein KT (2001). Das Klima. Bezirkskunde Osttirol.

[33] Netting RM (1981). Balancing on an Alp - Ecological Change & Continuity in a Swiss Mountain Comunity.

[34] Vogl-Lukasser B, Vogl CR, Reiner H (2007). The turnip (Brassica rapa L. subsp. rapa) in Eastern Tyrol (Lienz district; Austria). Ethnobot Res Appl.

[35] Mair W, Lehrerverein KT (2001). Osttirols Bergwelt - ein Steiflicht. Bezirkskunde Osttirol.

[36] Waschgler H, Lienz KTLL (1993). Landeskunde. Bezirkskunde Osttirol.

[37] Webpage of Sillian [http://www.marktgemeinde-sillian.at/]. Accessed 23 Aug 2018.

[38] Bevölkerungsentwicklung [http://www.statistik-austria.at/web_de/statistiken/menschen_und_gesellschaft/bevoelkerung/index.html]. Accessed 23 Aug 2018.

[39] Statistik-Austria (2018). Land- und Forstwirtschaft.

[40] Bye R, Ramamoorthy TP, Bye R, Lot A, Fa J (1993). The role of humans in the diversification of plants in Mexico. Biological diversity of Mexico - origins and distribution.

[41] Vogl-Lukasser B, Vogl CR, Gütler M, Heckler S (2010). Plant species with spontaneous reproduction in homegardens in eastern Tyrol (Austria): perception and management by women farmers. Ethnobot Res Appl.

[42] Reyes-García V, Vila S, Aceituno-Mata L, Calvet-Mir L, Garnatje T, Jesch A, Lastra JJ, Parada M, Rigat M, Vallès J, Pardo-de-Santayana M (2010). Gendered Homegardens: a study in three mountain areas of the Iberian Peninsula. Econ Bot.

[43] Erhardt W, Götz E, Bödeker N, Seybold S (2014). Zander: Handwörterbuch der Pflanzennamen.

[44] Gbedomon RC, Assogbadjo AE, Salako VK, Fandohan AB, Glèlè Kakaï R (2016). Exploring the spatial configurations of home gardens in Benin. Sci Hortic.

[45] Cruz-Garcia GS, Struik PC (2015). Spatial and seasonal diversity of wild food plants in home gardens of Northeast Thailand. Econ Bot.

[46] Abebe T, Bongers F (2012). Land-use dynamics in enset-based agroforestry homegardens in Ethiopia. Forest-people Interfaces: Understanding Community Forestry and Biocultural Diversity.

[47] Abebe T, Sterck FJ, Wiersum KF, Bongers F (2013). Diversity, composition and density of trees and shrubs in agroforestry homegardens in Southern Ethiopia. Agrofor Syst.

[48] Junqueira AB, Souza NB, Stomph TJ, Almekinders CJM, Clement CR, Struik PC (2016). Soil fertility gradients shape the agrobiodiversity of Amazonian homegardens. Agric Ecosyst Environ.

[49] Calvet-Mir L, Calvet-Mir M, Vaqué-Nuñez L, Reyes-García V (2011). Landraces in situ conservation: a case study in high-mountain home gardens in Vall Fosca, Catalan Pyrenees, Iberian Peninsula^1^. Econ Bot.

[50] Sunwar S, Thornström CG, Subedi A, Bystrom M (2006). Home gardens in western Nepal: opportunities and challenges for on-farm management of agrobiodiversity. Biodivers Conserv.

[51] Vlkova M, Polesny Z, Verner V, Banout J, Dvorak M, Havlik J, Lojka B, Ehl P, Krausova J (2011). Ethnobotanical knowledge and agrobiodiversity in subsistence farming: case study of home gardens in Phong My commune, central Vietnam. Genet Resour Crop Evol.

[52] Mellisse BT, van de Ven GWJ, Giller KE, Descheemaeker K. Home garden system dynamics in Southern Ethiopia. Agrofor Syst. 2017:1–17.

[53] Mwavu EN, Ariango E, Ssegawa P, Kalema VN, Bateganya F, Waiswa D, Byakagaba P (2016). Agrobiodiversity of homegardens in a commercial sugarcane cultivation land matrix in Uganda. Int J Biodivers Sci Ecosyst Serv Manag.

[54] Reyes-García V, Aceituno L, Vila S, Calvet-Mir L, Garnatje T, Jesch A, Lastra JJ, Parada M, Rigat M, Vallès J, Pardo-De-Santayana M (2012). Home gardens in three mountain regions of the Iberian Peninsula: description, motivation for gardening, and gross financial benefits. J Sustain Agric.

[55] Sharangi AB (2013). Spices not just spicy: role in human health with medicinal and therapeutic potentialities. Advances in Food Science and Technology.

[56] Srinivasan K (2005). Role of spices beyond food flavoring: nutraceuticals with multiple health effects. Food Rev Int.

[57] Pieroni A, Quave CL, Pieroni A, Price LL (2006). Functional foods or food medicines? On the consumption of wild plants among Albanians and southern Italians in Lucania. Eating and healing traditional food as medicine.

[58] Pieroni A (2000). Medicinal plants and food medicines in the folk traditions of the upper Lucca Province, Italy. J Ethnopharmacol.

[59] Huai H, Xu W, Wen G, Bai W (2011). Comparison of the Homegardens of eight cultural groups in Jinping County, Southwest China. Econ Bot.

[60] Gbedomon RC, Salako VK, Adomou AC, Glèlè Kakaï R, Assogbadjo AE (2017). Plants in traditional home gardens: richness, composition, conservation and implications for native biodiversity in Benin. Biodivers Conserv.

[61] Gbedomon RC, Salako VK, Fandohan AB, Idohou AFR, Glèlè Kakaï R, Assogbadjo AE (2017). Functional diversity of home gardens and their agrobiodiversity conservation benefits in Benin, West Africa. J Ethnobiol Ethnomed.

